# The association between lifelong personality and clinical phenotype in the FTD-ALS spectrum

**DOI:** 10.3389/fnins.2023.1248622

**Published:** 2023-10-04

**Authors:** Giulia Vinceti, Chiara Carbone, Chiara Gallingani, Luigi Fiondella, Simone Salemme, Elisabetta Zucchi, Ilaria Martinelli, Giulia Gianferrari, Manuela Tondelli, Jessica Mandrioli, Annalisa Chiari, Giovanna Zamboni

**Affiliations:** ^1^Department of Biomedical, Metabolic and Neural Sciences, University of Modena and Reggio Emilia, Modena, Italy; ^2^Neurology Unit, Ospedale Civile Baggiovara, Azienda Ospedaliero Universitaria di Modena, Modena, Italy; ^3^Department of Primary Care, Azienda Unità Sanitaria Locale di Modena, Modena, Italy

**Keywords:** frontotemporal dementia, amyotrophic lateral sclerosis, personality, premorbid, presymptomatic, disease phenotype, functional connectivity, Salience network

## Abstract

**Introduction:**

Frontotemporal dementia (FTD) and amyotrophic lateral sclerosis (ALS) are two phenotypes of the same neurodegenerative disease, the FTD-ALS spectrum. What determines the development of one rather than the other phenotype is still unknown. Based on the clinical observation that patients’ personality seems to differ between the two phenotypes, i.e., ALS patients tend to display kind, prosocial behaviors whereas FTD patients tend to present anti-social behaviors, and that these traits are often reported as pre-existing the disease onset by caregivers, we set up to study experimentally patients’ personality in their premorbid life.

**Methods:**

We first tested for differences between groups, then tested the association between premorbid personality and current functional organization of the brain. Premorbid personality of a cohort of forty patients, 27 FTD and 13 ALS, was explored through the NEO Personality Inventory 3 (NEO-PI-3), which analyses the five main personality factors, completed by the caregiver with reference to patient’s personality 20 years before symptoms onset (premorbid). A subgroup of patients underwent a brain MRI including structural and resting-state functional MRI (rsfMRI).

**Results:**

A significant difference between FTD and ALS in premorbid personality emerged in the Openness (133.92 FTD vs. 149.84 ALS, *p* = 0.01) and Extraversion (136.55 FTD vs. 150.53 ALS, *p* = 0.04) factors. This suggests that ALS patients had been, in their premorbid life, more open to new experiences, more sociable and optimistic than FTD patients. They also showed greater functional connectivity than both FTD and a control group in the Salience resting state network, over and above differences in gray matter atrophy. Finally, there was a positive correlation between premorbid Openness and functional connectivity in the Salience network across all patients, suggesting a possible association between premorbid personality and current functional organization of the brain, irrespective of the degree of atrophy.

**Discussion:**

Our proof-of-concept results suggest that premorbid personality may eventually predispose to the development of one, rather than the other, phenotype in the FTD-ALS spectrum.

## 1. Introduction

Frontotemporal dementia (FTD) and amyotrophic lateral sclerosis (ALS) are now considered to be two clinical expressions of the same neurodegenerative disease, the FTD-ALS spectrum (Neary and Snowden, 2013). An association between the two diseases was first observed as early as in the 1990s (Neary et al., 1990). The subsequent discovery of convergent genetic and pathological substrates reinforced the notion that the two diseases are different manifestations of a continuum ranging from pure forms of ALS with exclusive motor involvement, to pure forms of FTD, of which the most frequent presentation is behavioral (bvFTD), with exclusive cognitive/behavioral involvement, passing through hybrid forms of FTD-ALS with both cognitive and motor involvement (Burrell et al., 2016).

The population of susceptible neurons differs substantially in ALS and FTD, involving, respectively, upper/lower motor neurons and prefrontal/insular/temporal neurons. The clinical phenotype best reflects the specific pattern of neuronal loss. Nonetheless, ubiquitin-positive intracellular inclusions were initially found as the most frequent neuropathological correlate in susceptible regions in both diseases, and lately the phosphorylated form of TAR-DNA binding protein (TDP-43) was recognized as the principal compound of these ubiquitinated inclusions, pointing to a common pathological mechanism for FTD and ALS (Neumann et al., 2006).

Meanwhile, for many decades it has been observed that there are familiar clusters of FTD, ALS and FTD-ALS. Associations with loci on chromosome 17 and 9q and subsequent causative genes for both diseases were identified (*TARDBP* gene, *FUS* gene), leading to the pivotal discovery of the chromosome 9 open reading frame 72 (*C9orf72*) hexanucleotide GGGGCC repeat expansion in 2011 (DeJesus-Hernandez et al., 2011; Renton et al., 2011), which was rapidly recognized as the major cause of both familial FTD and familial ALS, and the most frequent mutation associated with FTD-ALS cases.

The knowledge about the clinical variability of the FTD-ALS syndromic complex and the wide range of the involved genetic and pathological factors has grown enormously over the past decade (Meeter et al., 2017). However, what affects the development of one rather than the other clinical phenotype remains substantially unknown. Some authors have found associations between specific neuroinflammatory profiles and the different clinical phenotypes (Oeckl et al., 2019), but no predisposing factors have been identified yet.

In recent years, the concepts of resilience and vulnerability to neuropathology have become increasingly crucial to the understanding of the interindividual variability in the brain response to pathology in neurodegenerative diseases. As an example, it has been largely shown that subjects with Alzheimer’s disease (AD) with higher education and level of occupation are resilient to neuropathology, i.e., they require more brain damage to exhibit the same level of clinical decline of less educated patients (Stern et al., 1994). This concept has been indicated as *reserve* and framed in terms of anatomical reserve (*brain reserve*, i.e., amount of brain tissue) and functional reserve (*cognitive reserve*, i.e., neural networks integrity and efficiency). Conversely, there are conditions which seem to confer greater vulnerability to the development of specific neurological syndromes. As an example, it has been shown that patients with the logopenic variant of primary progressive aphasia have a significantly higher incidence of anamnestic dyslexia than the healthy reference population (Rogalski et al., 2014). This suggests that they may have greater vulnerability to the dysfunction of the parieto-temporal language areas, thus manifesting aphasia symptoms earlier than other subjects with the same degree of pathological insult (Miller et al., 2013). Similarly, patients with posterior cortical atrophy (PCA) seem to have higher prevalence of mathematical and visuospatial learning disabilities than those with other forms of dementia and the general population (Miller et al., 2018). All these observations suggest that the way in which the brain has been used throughout one’s life may be associated with specific clinical phenotypes once the brain is hit by later-life neurodegenerative diseases.

In the last few years, the idea that individual personality may play a role in the development and progression of different diseases, including neurodegenerative diseases, has gained increasing attention (Low et al., 2013; Rouch et al., 2019). Personality can be defined as a set of constant patterns of perceiving, relating, and thinking about the environment and oneself, which the subject exhibits in a wide range of social and personal contexts (American Psychiatric Association [APA], 2014). In clinical practice it has been anecdotally observed that patients with ALS and bvFTD have some recurrent features in their personality, which seem to have been stable and already present before the onset of the symptoms. Neurologists caring for patients with ALS have variably described them as “pleasant,” “friendly”, “sympathetic”, “nice” (Wilbourn and Mitsumoto, 1998; Borasio and Miller, 2001; Mehl et al., 2017), emphasizing their active involvement in medical care and their remarkable resilience in coping with their disease. Indeed, since the 1970s several authors have described the peculiar personality profile of patients with ALS in comparison with either healthy controls or patients with other chronic, progressive non-neurodegenerative conditions, suggesting a possible relationship with the etiological factors of the disease (Brown and Mueller, 1970; Peters et al., 1978; Grossman et al., 2006; Mehl et al., 2017; Parkin Kullmann et al., 2018).

Conversely, neurologists caring for patients with the bvFTD are frequently told by the caregivers that the patient’s personality has always been scarcely pro-social and aversive along all their life, long before they developed the behavioral disturbances typical of this clinical phenotype, which include disinhibition, loss of manner, socially inappropriate and impulsive behaviors, and emotional bluntness. However, the recurrent clinical observation that ALS and bvFTD patients are often characterized by strikingly different personalities, has never been tested experimentally. Their *premorbid* personality has never been directly compared.

In the present study we systematically investigated the premorbid features of patients with ALS and bvFTD with the hypothesis that personality itself may play a role in modulating the phenotypic expression of the neuropathologic process along the FTD-ALS spectrum. We questioned if some specific individual pre-morbid personality factors constitute a *locus minoris resistentiae* thus conferring greater vulnerability or, on the contrary, a strength that influences the type of symptomatic manifestation once the neurodegenerative process begins. We first tested if patients with ALS and bvFTD have different premorbid personalities, through the administration to the caregivers of personality inventories with reference to how the patient had been in the past. We then investigated if the variability of premorbid personality modulated the functional organization of the brain in the present, i.e., once the neurodegenerative process has begun to cause symptoms, over and above the atrophy due to the neurodegenerative process itself. We reasoned that a premorbid anti-social attitude may confer vulnerability to the development of the cognitive-behavioral, rather than the motor, form of the disease (bvFTD). Conversely, a robust pro-social and empathic premorbid personality profile may confer resiliency to the cognitive-behavioral form in favor of the motor manifestation of the disease (ALS).

## 2. Materials and methods

### 2.1. Participants

Consecutive, eligible bvFTD and ALS patients seen at the Cognitive and Motor Neuron Disease Clinics of the Neurology Unit, Azienda Ospedaliero Universitaria di Modena in the period January 2018–October 2021 were prospectively recruited. Eligibility was defined based on the following inclusion criteria: a diagnosis of bvFTD, and/or a diagnosis of ALS according to existing diagnostic criteria (Brooks et al., 2000; Gorno-Tempini et al., 2011; Rascovsky et al., 2011), and presence of a caregiver who had known the patient for at least 20 years. We purposefully decided not to include patients with language presentations of FTD because, in our clinical experience, we have not noticed common traits of personality in patients with primary progressive aphasia as we have for bvFTD patients. Exclusion criteria consisted of a diagnosis of stroke, head trauma, epilepsy, or neurodegenerative disease other than FTD and ALS. Each patient underwent a cognitive and behavioral assessment. A subset of patients underwent genetic testing for mutations known to be associated to the FTD-ALS spectrum. Patients who did not have contraindications also underwent an MRI scan. Patients and caregivers were asked to fill in questionnaires to evaluate patients’ premorbid and current personality. The study was conducted under ethical approval of the Local Ethics Committee (Number 247/18) and all subjects gave written informed consent before recruitment. For the purpose of the imaging comparisons only, imaging data from a group of 10 healthy volunteers from a different study, also approved by the Local Ethics Committee (Number 134/14), were included as control group.

### 2.2. Clinical and neuropsychological assessment

Demographic and clinical data were collected for all patients, as well as neuropsychological data assessed with a standardized battery of cognitive tests, which included, among the others, the Mini-Mental State Examination (MMSE), California Verbal Learning Test (CVLT), Number Location Test from the Visual Object Space Perception Battery (VOSP), Modified-Trails Making Test (M-TMT), Modified Five-Point Test (MFPT), M&N Alternation, F-words per minute, Couples of words and Proverb Interpretation, Calculation, Stroop Test, and Digit Span, abbreviated Boston Naming Test (15 items BNT), Peabody Picture Vocabulary Test revised (PPVT-R), syntax comprehension subtest of the Curtiss-Yamada Comprehensive Language evaluation-Receptive test (CYCLE-R), word reading section of the Wide Range Achievement test-4 (WRAT-4) and reading of irregular words to evaluate single-word reading and surface dyslexia, multiple repetitions of multisyllabic words and repetition of sentences from the Motor Speech Evaluation (MSE). Behavioral symptoms were assessed through the Neuropsychiatric Inventory (NPI) (Cummings, 1997), and depression symptoms with the Beck Depression Inventory-II (BDI-II) (Ghisi and Sanavio, 2006).

### 2.3. Personality evaluation

Patients’ personality was assessed with NEO Personality Inventory 3 (NEO-PI-3) (McCrae et al., 2005), which is the most commonly used questionnaire in the scientific literature to describe personality according to the Five-Factor Model (FFM), the most prominent classification system of personality. According to the FFM, personality results from the combination of five key traits, often called the “Big Five,” which include Neuroticism, Extraversion, Openness, Agreeableness, and Conscientiousness (Digman, 1990). Each dimension can be further divided in six facets, that allow for a more refined analysis.

In its latest version, the NEO-PI-3 consists of 240 items which are statements describing various situations of daily life: the respondent must answer to each item on a five-point Likert scale (1 = completely disagree; 5 = completely agree). It is adapted in an Italian version and takes 30–40 min to be completed.

The analysis of all the responses combined allows to obtain a score for each of the five dimensions – Neuroticism, Extraversion, Openness, Agreeableness and Conscientiousness–and the thirty subcategories or facets. Altogether, they provide a detailed picture of subject’s personality. There is not a cut-off of normality for each score, but the higher the score, the stronger the representation of that dimension in the subject’s personality. Country-specific normative data are provided in the NEO-PI-3 Manual. The Italian neutral reference sample (RS) includes 727 individuals.

For the purposes of the present study, we administered the NEO-PI-3 to the patient’s caregiver twice and asked them to complete the questionnaire with reference to the patient’s personality at two timepoints: (i) referring to the patient’s current situation (after disease onset, i.e., current personality) and (ii) referring to how the patient had been 20 years before (before disease onset, i.e., premorbid personality). In a setting of patients with variable degrees of cognitive impairment, behavioral disturbances, and poor insight, caregivers’ compilation is essential, especially for FTD patients, as self-administered questionnaires would have not been equally reliable across patients.

### 2.4. Statistical analysis

Analysis of demographic, neuropsychological and behavioral data were performed with Stata 16.1. Parametric and non-parametric analysis, as appropriate, were made for comparisons between diagnostic groups. A *p*-value < 0.05 was considered statistically significant. Differences in personality factors between groups at each timepoint were tested with repeated-measures ANOVA. Analysis of association between variables were performed with Pearson’s correlation.

### 2.5. Imaging analysis

Patients underwent a multimodal MRI protocol at the Ospedale Civile Baggiovara, Azienda Ospedaliero Universitaria di Modena on a 3T GE scanner equipped with a 48-channel-array head coil. The imaging protocol included, among other sequences, high-resolution T1-weigthed 3D BRAVO structural images (TR 2.15 s; TE 3.1 ms; FOV 328 × 512 × 340; voxel dimension 1 mm isotropic) and single-shot gradient echoplanar imaging (EPI) T2*-weighted images acquired along the transverse plane, parallel to the anterior to posterior commissural line, while the subject rested for fMRI (TR 1.7 s; TE 31.0 ms; slice thickness 3 mm including a 0.3 mm gap; voxel dimension 3 mm isotropic; 200 volumes). Images were analyzed using FSL (FMRIB Software Library) v6.0 software.^1^ Structural data were analyzed with FSL-VBM (Douaud et al., 2007), an optimized voxel-based morphometry (VBM) protocol (Good et al., 2001) carried out with FSL tools (Smith et al., 2004). First, single-subject structural images were brain-extracted and gray matter-segmented before being registered to the MNI 152 standard space using non-linear registration (Andersson et al., 2007). The resulting images were averaged and flipped along the x-axis to create a left-right symmetric, study-specific gray matter template. Second, all native gray matter images were non-linearly registered to this study-specific template and “modulated” to correct for local expansion (or contraction) due to the non-linear component of the spatial transformation. The modulated gray matter images were then smoothed with an isotropic Gaussian kernel with a sigma of 3 mm. Voxelwise General Linear Modeling (GLM) was applied using permutation-based non-parametric testing (*randomise* command in FSL, with 5,000 permutations), correcting for multiple comparisons across space, to perform comparison analyses exploring differences in GM volumes between groups (FTD, ALS, and controls). Age of patients was also mean-centered and entered as covariate of no interest to control for its potential effect.

Resting state fMRI data were analyzed using probabilistic independent component analysis (ICA) as implemented in the Multivariate Exploratory Linear Optimized Decomposition into Independent Components FSL tool (MELODIC) (Beckmann and Smith, 2004; Beckmann et al., 2005). Noise components were manually classified using criteria developed by Griffanti et al. (2017), and denoised data of all patients were temporally concatenated and decomposed into 25 components [in line with previous studies (Zamboni et al., 2013)] using ICA to identify large-scale networks of covariation during rest. Pre-processed and denoised functional data from an equal number of participants randomly selected in each group (controls, FTD, and ALS) were used for this purpose to avoid bias toward the larger group (the total number of data sets included was 21). The concatenated fMRI data sets were decomposed using ICA to identify large-scale patterns of functional connectivity in the whole sample. Eight biologically valid, non-artfactual resting state networks (RSNs) were identified both by visual inspection and by using spatial correlation against a set of previously defined maps. Dual regression was then used to generate subject-specific versions of the RSN maps (Filippini et al., 2009). We performed group comparisons on functional connectivity in the Default mode network (DMN), two sensory-motor networks (one more anterior and one more posterior), and the Salience network (SN), which we had hypothesized to be different between the groups based on previous literature.

Voxel-wise statistics were then performed using *randomise* in FSL (with 5,000 permutations) to compare diagnostic groups on the RSNs on which we had *a priori* hypothesis, i.e., those involved in social cognition and executive functions (Salience, fronto-parietal, Default mode RSNs) and motor control (sensory–motor RSN), plus a control network on which we did not expect significant changes (visual RSN). Gray matter values obtained from VBM were entered in all the GLM as covariate of no interest to control for the potential effects of regional atrophy on fMRI comparisons. Results were considered significant at *p* < 0.05, fully corrected with threshold-free cluster enhanced (TFCE) correction. After observing significant results, parameter estimates of functional connectivity were extracted from the significant resulting regions (functional ROIs) and from the whole RSN of interest and correlated with premorbid NEO-PI-3 scores.

### 2.6. Data availability

Anonymized data will be made available upon request and permission granted by our Local Ethics Committee.

## 3. Results

### 3.1. Sample clinical and neuropsychological characteristics

Forty consecutive patients (mean age 68.1 years, 19 females), along with their caregivers, were recruited. Among them, 27 had a diagnosis of bvFTD and 13 had a diagnosis of ALS. Three patients of the first group later developed ALS too. Genetic data for FTD-ALS spectrum mutations was available for 14 patients and resulted in 1 *MAPT* gene mutation (a bvFTD patient with positive family history for dementia) and 4 *C9orf72* repeat expansions (2 bvFTD-ALS both with positive family history for FTD and ALS, 1 bvFTD with no family history for neurodegenerative/psychiatric disease, 1 ALS with positive family history for ALS and FTD).

Clinical and neuropsychological profile of the FTD and ALS group, and their comparison, are reported in Table 1.

**TABLE 1:** Demographic and neuropsychological characteristics of patients.

	FTD	ALS	FTD vs. ALS
**Demographic characteristics**			
Number	27	13	–
Gender M:F	18:9	8:5	*p* = 0.424
Age (y)	69.5 (10.5)	63.4 (11.2)	*p* = 0.054
Education (y)	10.21 (4.68)	11.50 (3.60)	*p* = 0.196
Disease duration (y)	5.38 (3.49)	2.30 (3.12)	*p* < 0.001*
**Neuropsychological characteristics**			
MMSE	24.48 (3.73)	27.80 (3.19)	*p* = 0.010**
CVLT free recall (10’)	3.46 (2.40)	7.50 (0.93)	*p* < 0.001**
VOSP	6.92 (3.27)	9.33 (0.87)	*p* = 0.073
M-TMT correct lines	8.39 (5.63)	14 (0)	*p* = 0.008**
MFPT correct figures	4.68 (3.08)	7 (5.51)	*p* = 0.281
MFPT repeated figures	3.82 (5.74)	1.29 (1.25)	*p* = 0.479
M&N Alternation	1.07 (0.90)	0 (0)	*p* = 0.008*
Abstraction	2.12 (1,56)	4.78 (1,20)	*p* < 0.001**
Calculation	3.21 (1.73)	4.50 (0.76)	*p* = 0.064
Stroop 3	23.85 (12.71)	56.50 (6.95)	*p* < 0.001**
Direct span	4.93 (1.02)	5.78 (1.20)	*p* = 0.068
Reverse span	3.07 (1.15)	4.56 (1.33)	*p* = 0.008**
BNT	10.58 (2.44)	13.56 (1.33)	*p* = 0.001**
Phonemic fluency	8.08 (3.95)	13.88 (5.87)	*p* = 0.008**
Semantic fluency	10.04 (4.46)	21.13 (4.26)	*p* < 0.001**
PPVT-R	12.38 (2.30)	14.33 (0.71)	*p* = 0.007**
CYCLE-R	3.96 (1.40)	4.89 (0.33)	*p* = 0.093
Reading	72.32 (5.48)	75.56 (0.53)	*p* = 0.009**
Verbal agility	4 (1.36)	3.33 (1.66)	*p* = 0.382
Repetition	4.15 (0.99)	4.89 (0.33)	*p* = 0.026**
CATS face concordance	9.69 (1.99)	12 (0)	*p* < 0.001**
CATS emotions recognition	9.62 (3.66)	13.33 (0.87)	*p* = 0.004**
NPI	25.72 (14.85)	3.60 (4.98)	*p* < 0.001*
BDI-II	14.91 (11.36)	12.38 (10.17)	*p* = 0.717

Reported values are means with standard deviation values in parenthesis. The last column on the right reports the results of the comparison between diagnostic groups (level of statistical significance *p* < 0.05). *FTD > ALS, **ALS > FTD. MMSE, Mini-Mental State Examination; CVLT, California Verbal Learning Test; VOSP, Visual Object Space Perception Battery; M-TMT, Modified-Trails Making Test; MFPT, Modified Five-Point Test; BNT, Boston Naming Test; PPVT-R, Peabody Picture Vocabulary Test revised; CYCLE-R, Curtiss-Yamada Comprehensive Language evaluation-Receptive test; WRAT-4, Wide Range Achievement test-4; CATS, Comprehensive Affect Testing System; NPI, Neuropsychiatric Inventory; BDI-II, Beck Depression Inventory.

The groups did not differ significantly in terms of sex balance and education. There was almost a significant difference in age, with FTD patients older than ALS patients (69.5 vs. 63.4 years, *p* = 0.054). Also, FTD patients showed a longer disease duration (5.38 vs. 2.30 years; *p* < 0.001) at enrolment.

Amyotrophic lateral sclerosis patients performed better than FTD in most cognitive tests, consistently with the fact that they did not complain of major cognitive deficits. The only exception was the verbal agility task, in which FTD patients performed slightly better than ALS patients (4 vs. 3.33, *p* = 0.382), probably because of dysarthria in ALS patients.

As expected, patients with FTD had significantly more behavioral disturbances than patients with ALS as measured with NPI (25.72 vs. 3.60; *p* < 0.001).

Both groups showed low scores for depression at BDI-II. The median score for ALS patients was slightly lower than for FTD patients (12.38 vs. 14.91).

### 3.2. Analysis of personality profiles

Tables 2, 3 show the five domains’ scores for the two patient groups as well as for the Italian reference sample (RS) at the two different timepoints: before disease onset (premorbid personality) and after disease onset (current personality).

**TABLE 2:** Premorbid personality profile in the FTD and ALS groups.

	FTD (*n* 27)	ALS (*n* 13)	RS (*n* 727)	FTD vs. RS (*p*)	ALS vs. RS (*p*)	FTD vs. ALS (*p*)
**Premorbid personality**						
Neuroticism	126.88 (22.69)	127.53 (22.08)	136.19 (22.10)	0.04*	0.18	0.93
Extraversion	136.55 (22.29)	150.53 (11.34)	152.69 (20.37)	<0.001*	0.50	0.04*
Openness	133.92 (18.46)	149.84 (18.10)	162.66 (23.07)	<0.001*	0.02*	0.01*
Agreeableness	160.40 (23.17)	163.38 (16.44)	156.64 (16.89)	0.40	0.16	0.68
Conscientiousness	166.81 (27.41)	174.76 (17.78)	162.21 (23.66)	0.39	0.02*	0.34

Reported values are means with standard deviation values in parenthesis, and are shown for FTD, ALS and the reference sample (RS). The last three columns on the right report the results of the comparison between each diagnostic groups (FTD and ALS) and the RS, as well as between FTD and ALS, respectively (level of statistical significance *p* < 0.05).

**TABLE 3:** Current personality profile in the FTD and ALS groups.

	FTD (*n* 27)	ALS (*n* 13)	RS (*n* 727)	FTD vs. RS (*p*)	ALS vs. RS (*p*)	FTD vs. ALS (*p*)
**Current personality**						
Neuroticism	160.40 (23.17)	163.38 (16.44)	136.19 (22.10)	0.15	0.28	0.68
Extraversion	119.54 (28.40)	132.36 (25.46)	152.69 (20.37)	<0.001*	0.02*	0.21
Openness	127.09 (21.63)	142.09 (21.33)	162.66 (23.07)	<0.001*	0.009*	0.06
Agreeableness	158.45 (16.34)	172.90 (15.33)	156.64 (16.89)	0.60	0.005*	0.02*
Conscientiousness	142.68 (29.68)	166.27 (33.31)	162.21 (23.66)	0.005*	0.69	0.04*

Reported values are means with standard deviation values in parenthesis, and are shown for FTD, ALS and the reference sample (RS). The last three columns on the right report the results of the comparison between each diagnostic groups (FTD and ALS) and the RS, as well as between FTD and ALS, respectively (level of statistical significance *p* < 0.05).

**FIGURE 1:**
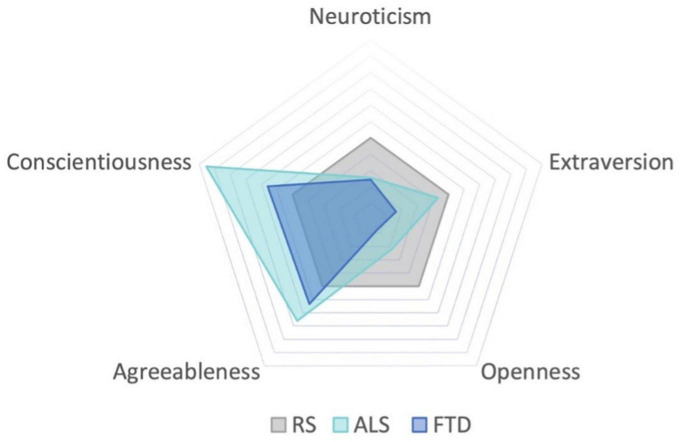
Comparison of premorbid personality profile of ALS and FTD, with reference to the RS.

When comparing premorbid personality (Figure 1 and Table 2), a significant difference between groups emerged in the Extraversion domain, with ALS scoring higher than FTD (150.53 vs. 136.55, *p* = 0.04). Analyses of the facets underlying the Extraversion domain showed that group differences in premorbid Extraversion were mainly driven by the “Positive emotions” facet, which was higher in ALS compared to FTD (27.30 vs. 23.29, *p* = 0.013, see Supplementary Table 1).

The Openness domain also revealed a significant difference between groups, again with ALS scoring higher than FTD patients (149.84 vs. 133.92, *p* = 0.01). Differences in premorbid Openness were mainly driven by the facets “Openness to Fantasy” (21.07 vs. 24.92, *p* = 0.031) and “Openness to Feelings” (24.22 vs. 27.46, *p* = 0.031), which resulted significantly lower in FTD compared to ALS (see Supplementary Table 1). No other significant differences in premorbid personality emerged in the remaining domains (Neuroticism, Agreeableness, Consciousness).

Importantly, all patients with gene mutations (1 *MAPT* bvFTD patient, 2 *C9orf72* bvFTD-ALS patients, 1 *C9orf72* bvFTD patient and 1 *C9orf72* ALS patient) presented premorbid personality profiles which were close to the mean of the corresponding clinical group, there not standing as outliers.

When comparing premorbid personality of each patient group (FTD and ALS) to the mean of the reference sample (RS) of the Italian population (727 subjects), we found some relevant differences. FTD scored low in Neuroticism (126.88 vs. 136.19, *p* = 0.04), Extraversion (136.55 vs. 152.69, *p* < 0.001) and Openness (133.92 vs. 162.66, *p* < 0.001). ALS scored low in Openness (149.84 vs. 162.66, *p* = 0.02), while they scored high in Conscientiousness (174.76 vs. 162.21, *p* = 0.02).

When comparing current personality (Table 3), the difference between ALS and FTD in the Openness domain persisted with a trend to statistical significance (142.09 vs. 127.09, *p* = 0.06), while the difference in Extraversion domain disappeared. In addition, groups comparisons of current personality showed the emerging of a significant difference between ALS and FTD in both Agreeableness (172.90 vs. 158.45, *p* = 0.02) and Conscientiousness (166.27 vs. 142.68, *p* = 0.04). Comparisons with the RS showed that FTD scored low in Extraversion (119.54 vs. 152.69, *p* < 0.001), Openness (127.09 vs. 162.66, *p* < 0.001), and Conscientiousness (142.68 vs. 162.21, *p* = 0.005) at present time. Conversely, ALS scored low in Openness (142.09 vs. 162.66, *p* = 0.009) and Extraversion (132.36 vs. 152.69, *p* = 0.02) and high in Agreeableness (172.90 vs. 156.64, *p* = 0.005).

Repeated measures ANOVAs performed including premorbid and current scores to study the effect of time over personality (see Supplementary Result section, and Supplementary Figures 1–5) showed a significant effect of time on both Extraversion (*p* = 0.027) and Openness (*p* = 0.004), in the sense that current scores in these two domains had further decreased in both FTD and ALS patients relative to premorbid scores. A significant interaction between time and diagnostic group (*p* = 0.02) in the Agreeableness domain suggested that current Agreeableness scores had decreased in FTD and increased in ALS patients relative to premorbid scores. Finally, there was an almost significant interaction between time and diagnostic group (*p* = 0.06) in the Conscientiousness domain. In fact, both groups decreased their score during time, but while the reduction was mild for ALS patients, it appeared extremely marked for FTD patients.

### 3.3. Imaging comparisons

Twenty-eight out of 40 patients (21 FTD and 7 ALS) were able to undergo a brain MRI scan. The three patients with gene mutations included in the imaging analysis (2 bvFTD patients, one with MAPT mutation and one with *C9orf72* expansion, and 1 FTD-ALS patient with *C9orf72* expansion) had parameter estimates of Salience network functional connectivity comparable to the mean of the entire patient population.

A VBM comparison on GM density between the FTD, ALS and control groups did not give significant results (Supplementary Figure 6 reports the uncorrected results at *p* < 0.001). Voxel-wise, subject-specific maps of gray matter density obtained by the VBM were nevertheless included in the subsequent comparisons of functional connectivity as an additional covariate to control for the effect of atrophy and be sure that the functional results would not be simply driven by subjects’ different patterns of neurodegeneration.

**FIGURE 2:**
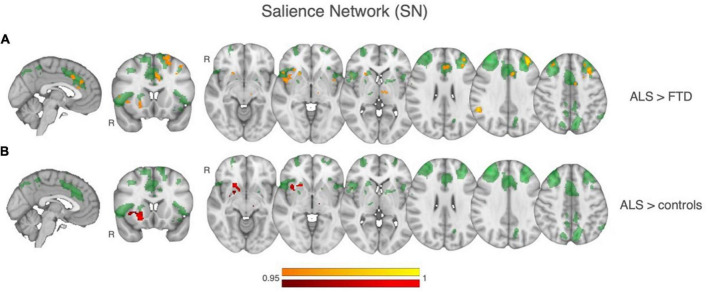
Results of between-group comparisons of rsfMRI connectivity within the Salience network (SN), shown in transparent green in all the images. **(A)** In yellow-orange, regions of significant greater SN functional connectivity in ALS relative to controls. **(B)** In red, regions of significant greater SN functional connectivity in ALS relative to FTD.

Group comparisons of functional connectivity within the identified resting state networks (RSNs) showed that patients with ALS had greater functional connectivity compared to FTD patients and the control group within the Salience network. Regions of greater functional connectivity in the Salience network in the ALS group relative to the FTD group were in the right insula, putamen, nucleus accumbens, and in the left thalamus (Figure 2). Regions of greater functional connectivity in the Salience network in the ALS group relative to the control group also included the left anterior cingulate cortex, left frontal pole and middle frontal gyrus, and right angular gyrus. Of note, the three patients with gene mutations included in the imaging analysis had parameter estimates of Salience Network functional connectivity comparable to the mean of the entire patient population. No significant differences were found between groups in any of all the other RSNs.

### 3.4. Association between premorbid personality and current functional connectivity

**FIGURE 3:**
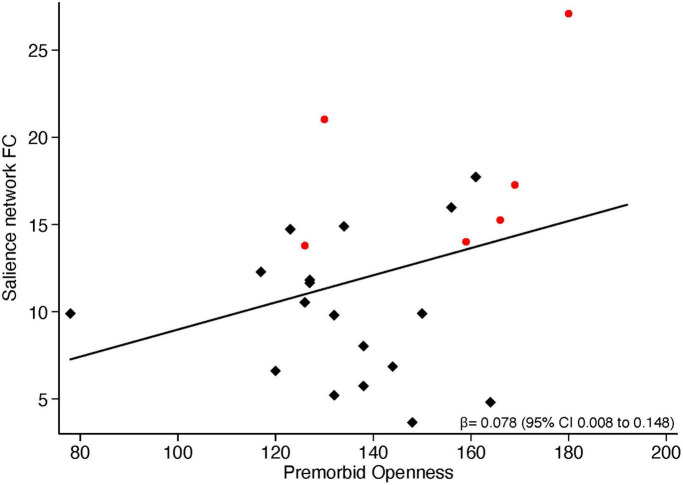
Correlation between functional connectivity in the Salience network and NEO-PI-3 premorbid Openness scores. Red dots represent ALS patients, black squares represent FTD patients.

Having found significant group differences between ALS and FTD patients in scores of premorbid Extraversion and premorbid Openness, as well as in the strength of current functional connectivity within the Salience network, we then explored if these two entities, i.e., premorbid personality and current functional connectivity, are associated. Across all patients (i.e., considering all patients altogether), a significant positive correlation emerged between parameter estimates of functional connectivity extracted from regions of significant difference between ALS and FTD in the Salience network and premorbid Openness (*r* = 0.37, *p* = 0.0312). A similar positive correlation (*r* = 0.358, *p* = 0.0372) emerged between the mean functional connectivity extracted from the whole Salience network and premorbid Openness (Figure 3).

A positive correlation at the trend level emerged between parameter estimates of functional connectivity extracted from the regions of significant difference between ALS and FTD in the Salience network and premorbid Extraversion (*r* = 0.30, *p* = 0.08).

A positive correlation was also found between mean parameter estimates of functional connectivity extracted from the whole Sensory-motor network and premorbid Openness (*r* = 0.32, *p* = 0.05).

## 4. Discussion

In this study we tested the hypothesis that, along the FTD-ALS continuum, patients with the two different phenotypes FTD, and ALS have different premorbid personality profiles. We found that ALS patients had higher scores relative to FTD patients in two domains of premorbid personality: Extraversion and Openness. We then found that ALS patients had greater functional connectivity in the Salience network relative to FTD patients, over and above differences in the pattern of atrophy. Importantly, premorbid personality scores and current functional connectivity significantly correlated, suggesting an association between lifelong personality traits and the subsequent functional response of the brain to the neurodegenerative damage.

Specifically, we found that FTD patients showed lower premorbid Extraversion and Openness scores compared to both ALS and to the reference sample. This means that individuals who subsequently developed the behavioral phenotype of the spectrum were originally characterized by a less open and extravert personality than individuals who subsequently developed the motor phenotype.

This is the key and novel finding of the present study. While only one previous study had investigated ALS patients’ premorbid personality, finding higher levels of Extraversion, Agreeableness, and Conscientiousness in ALS patients relative to healthy controls (Parkin Kullmann et al., 2018), no studies explored premorbid personality as a driving factor for phenotypic variability. Although in line with these results, our study shows that it is the FTD group, rather than the ALS, which deviates from the reference sample. Another study on current personality carried out in ALS patients detected lower scores in the Openness domain, as compared with patients with other chronic progressive non-neurologic diseases (Grossman et al., 2006). However, to the best of our knowledge, no studies directly compared premorbid personality between FTD and ALS patients.

The Extraversion personality trait captures qualities including sociability, assertiveness, and cheerfulness. In some ways, the introverted attitude might be best described as absence of Extraversion rather than its opposite. Conversely, individuals that are high in Extraversion are sociable: they like people and large gatherings. Extraverts tend to be assertive, active, and talkative. They like excitement and stimulation and tend to be cheerful in disposition. They are upbeat, energetic, and optimistic. Research on the cognitive neuroscience of Extraversion has gained much attention in the last decades, and several psychobiological theories of this domain have been formulated (Eysenck and Eysenck, 1985; Zuckerman, 2005; Corr, 2009), all sharing the assumption of the feed-forward model (Matthews, 2016). This model assumes that personality traits are encoded as a set of genetic polymorphisms in genes coding for brain development; then, the genetic influence, in conjunction with environmental factors, feed forward into brain functioning and behavior, leading to individual differences in brain functioning, which in turn generate individual differences in measured personality traits. Several theories identify the mesolimbic dopamine system as the primary mechanism underlying individual differences in Extraversion (Depue and Collins, 1999). Specifically, they implicate a neuroanatomical network and modulatory neurotransmitters in the processing of incentive motivation. This corticolimbic-striatal-thalamic network integrates the salient incentive context in the medial orbital cortex, amygdala, and hippocampus; it encodes the intensity of incentive stimuli in a motive circuit composed of the nucleus accumbens, ventral pallidum, and ventral tegmental area dopamine projection system and creates an incentive motivational state that can be transmitted to the motor system (Depue and Collins, 1999). Individual differences in the functioning of this network are thought to arise from functional variation in the ventral tegmental area dopamine projections, which are directly involved in coding the intensity of incentive motivation. Reward-reactivity has been linked with dopamine function in behavioral neuroscience research (Schultz, 1998; Robinson et al., 2005; Dalley et al., 2007) and with Extraversion in psychometric (Cooper et al., 2008) and behavioral research.

Also, several neuroimaging studies have been conducted to test the prediction of these theories. Among functional MRI studies, Cohen et al. (2005) showed that individual differences in Extraversion and the presence of the A1 allele on the dopamine D2 receptor gene predicted activation magnitudes in the brain’s reward system during a gambling task, fostering the link between personality, genetics, and brain functioning. Kennis et al. (2013) reviewed fMRI studies on the topic and concluded that Extraversion correlates consistently with increased activity in response to positive stimuli in several areas associated with dopaminergic pathways, including the ventral and dorsal striatum and ventral prefrontal cortex.

In our study, we found significant differences between ALS and FTD patients only in the Salience network (SN), which has been shown to be involved in the representation of the homeostatic relevance (i.e., salience) of ambient internal and external stimuli (Seeley, 2019). The anatomical distribution of the neurodegenerative process that gives rise to the FTD syndrome, particularly the behavioral variant (bvFTD), typically involves these tightly interconnected brain regions, which are the earliest regions to be involved by the neurodegenerative process, regardless of the underlying neuropathological etiology (Seeley et al., 2008; Perry et al., 2017). More precisely, we found that, within the Salience network, patients with ALS had a greater functional connectivity compared to FTD and to controls in the right insula, right putamen and right nucleus accumbens, and the left thalamus, over and above differences in atrophy. We then found that functional connectivity of these regions correlated with the degree of premorbid Extraversion and this was true for all the patients irrespective of the clinical phenotype. This finding creates a link between the behavioral difference in Extraversion trait found between ALS and FTD in premorbid personality and the current state of functional connectivity in the Salience network, that includes the corticolimbic-striatal-thalamic dopaminergic pathway so strongly connected to Extraversion in the literature.

Thus, we can speculate that an individual lifelong degree of Extraversion is associated with the brain functional organization, which in turn could influence the manifestation of a subsequent neurodegenerative process when occurring in the brain, by modulating the pattern of spreading of the disease along some preferential, already vulnerable neural circuits. More precisely, we can speculate that subjects with higher degrees of Extraversion might have become resilient to the bvFTD phenotype, because they had premorbidly developed stronger functional connectivity in regions of the Salience network that are also part of the mesolimbic pathway. Thus, when the neurodegenerative process begun, these subjects may have been paradoxically more vulnerable to the motor presentation of the disease, thus developing ALS. Interestingly, a recent study using computational modeling of structural connectivity networks in ALS showed that the networks that serve as conduits for the spread of regionally-preferential pathological transmission are not anchored in primary motor structures as commonly thought but in the basal ganglia, thalamus and insula (Pandya et al., 2022). These are the same critical regions of the Salience network that we found to have greater intrinsic functional connectivity in ASL patients compared to FTD and controls. Such areas may be the common sites involved in the very initial stages of the FTD-ALS continuum. Also, our finding that ALS displayed a more robust intrinsic functional connectivity at present time in these areas might be speculatively interpreted as compensatory response to the disease that pushes the pathological process to express prevalently in different (i.e., primary motor) areas.

We also found a significant difference in premorbid Openness between ALS and FTD.

Openness is a personality trait reflecting a broad range of cognitive–affective styles such as absorption in sensory experience, preference for novel experiences, curiosity, and creativity (McCrae et al., 2005). Open people are typically described as highly “permeable” and receptive to salient stimuli and strongly motivated to “enlarge” their sensory experience. At the brain level, some authors have proposed that Openness is associated with mesocortical networks (i.e., midbrain–prefrontal cortex (PFC) dopaminergic circuits) (DeYoung et al., 2010), and therefore both Openness and Extraversion have been associated with dopaminergic function. DeYoung et al. (2005) have also hypothesized that two distinct dopaminergic pathways underlie Openness and Extraversion: the mesocortical and mesolimbic pathways, respectively. It is therefore not surprising that we found similar results for both these domains.

We also found that the functional connectivity within the Salience network correlated with premorbid Openness, suggesting that also Openness could play a role in the modulation of the Salience network functional connectivity. Considering all these results, we could speculate that low levels of Openness, which seem to characterize both FTD and ALS, might represent a “risk factor” for the whole FTD-ALS spectrum of disease, and that different level of Openness could influence phenotypic manifestation of the disease, with very low degrees of Openness paving the way toward clinical phenotypes that originate from selective disruption of the Salience network key areas, i.e., FTD.

When looking at the current personality scores, the difference in Openness persisted also at present time, even if slightly attenuated. In the Extraversion domain, no significant difference appeared to persist at present time, since both groups displayed an important decrease in the domain’s scores, significantly low compared to the reference norm sample. Finally, differences between the two groups also appeared in current Agreeableness and Conscientiousness domains, and this could be partially explained in terms of adaptation to disease. For the Agreeableness domain, the difference in current personality was driven by the sharp increase in Agreeableness scores at present showed by ALS. Conversely, the marked difference in current Conscientiousness was driven by the sharp decline of FTD, which also ALS showed even if much more restrained. As expected, FTD patients appeared less pleasant after the development of dementia, as a symptom of the dementia itself. Actually, loss of empathy and disinhibition strictly characterize bvFTD, and they could be the effect of neurodegeneration directly impacting on the specific behavioral neurocircuits known to be selectively involved in the disease.

On the contrary, ALS patients manifested even higher level of kindness at the present moment compared to the premorbid state, which already showed scores in the upper normal reference range. This is in line to what experienced by those, clinicians or caregivers, who take care of ALS patients, who are often perceived as inexplicably resilient throughout their difficult disease and in spite of their inevitably poor prognosis, showing themselves collaborative to clinicians and prone to follow recommendations and treatment (Borasio and Miller, 2001). Again, these change along time in Agreeableness could be explained in term of response to neurodegeneration, which in this case could exacerbate an already present personality tract. A different longitudinal trajectory appeared to be followed by Conscientiousness domain, which in ALS group was characterized by absolute high scores in the past, that decreased over time and fitted in the normal range at the disease timepoint.

The present study has several limitations. The first relates to the small sample size, especially of the subsample of those who were able to undergo the MRI scan, and to the asymmetry between the numbers of FTD and ALS group. We shall therefore acknowledge that our results are exploratory and should be considered a proof-of-concept that will pave the road for future, prospective studies in larger cohorts. Another important limitation of the study is that personality evaluation of premorbid and current personality was based on caregivers’ ratings: this approach was chosen as an obliged alternative to self-compiled questionnaires, that in case of patients with variable degrees of cognitive impairment and insight could not be considered reliable. Nonetheless, caregivers’ evaluation is not immune to bias, since it could be less precise for recall difficulties or influenced by individual attitude and caring role itself. Also, caregivers were instructed to compile the “premorbid” form of the NEO-PI-3 referring to the patient 20 years before. However, we know that neurodegenerative diseases start many years before first symptoms, and even retrospectively it is not possible to date precisely when the disease started in a specific individual. So, we cannot be certain that the collected data on premorbid personality reliably reflect the premorbid state, thus indicating the need of studies with prospective design on this issue.

Also, the present study lacks a robust genetic analysis. We considered the pathogenetic mutations known to be associated with the disease (i.e., the FTD-ALS spectrum), which unfortunately were not available for all the patients. Conversely, we were not able to include analysis of potential genetic variants associated with personality traits in each different group of patients, to test for specific association between the genetic background of personality and clinical manifestation of the disease. Future studies should address this topic.

## 5. Conclusion

In this study, we found a significant difference in premorbid personality of FTD patients compared with ALS patients, the former being characterized by lower Extraversion and Openness many years before the disease onset. We then found differences in the current functional connectivity between ALS and FTD in a specific brain network, the Salience network, which includes key areas known to be associated with the Extraversion and Openness personality traits and that is specifically involved in FTD neurodegeneration. Finally, we found a correlation, across the whole group of patients, between functional connectivity in the Salience network and premorbid Extraversion and Openness, suggesting a possible association between premorbid personality and disease phenotype, i.e., ALS vs. bvFTD.

## Data availability statement

The raw data supporting the conclusions of this article will be made available by the authors, without undue reservation.

## Ethics statement

The studies involving humans were approved by the Comitato Etico Area Vasta Emilia Nord. The studies were conducted in accordance with the local legislation and institutional requirements. The participants provided their written informed consent to participate in this study.

## Author contributions

GV, JM, AC, and GZ designed the original study. GV, CC, CG, AC, and GZ analyzed the data and with the contribution of LF, SS, EZ, IM, GG, and MT interpreted the data and drafted the manuscript. All authors read and approved the final manuscript.

## Footnotes

1
https://fsl.fmrib.ox.ac.uk/fsl
