# Supplementary material for: The association between lifelong personality and clinical phenotype in the FTD-ALS spectrum
Source: Front Neurosci. 2023 Oct 4;17:1248622. doi: 10.3389/fnins.2023.1248622 (PMC10582748; doi:10.3389/fnins.2023.1248622)
Supplement: Supplementary file 1 [file Data_Sheet_1.docx]

**SUPPLEMENTARY RESULTS**

## Comparison between FTD and ALS patients’ *premorbid* personality: domains’ facets

The NEO-PI-3 scores for the five domains’ facets in premorbid personality were analyzed and are shown in Table 4. Regarding Extraversion, one facet, Positive emotions, was higher in ALS compared to FTD (27.30 vs 23.29, p=0.013); the facet Activity showed a trend of significance in the same direction (26.23 vs 22.74, p=0.061). The description of Activity implies that a high Activity score is seen in rapid tempo and vigorous movement, a sense of energy, and a need to keep busy; active people lead fast-paced lives. Conversely, low scorers are more leisurely and relaxed in tempo, though they are not necessarily sluggish or lazy.

In the Openness domain, two facets differed between FTD and ALS. Fantasy resulted significantly lower in FTD compared to ALS (24.92 vs 21.07, p=0.031). Individuals who are open to fantasy have a vivid imagination and an active fantasy life, they daydream not simply as an escape, but as a way of creating an interesting inner world for themselves. They elaborate and develop their fantasies and believe that imagination contributes to a rich and creative life. Low scorers are more prosaic and prefer to keep their minds on the task at hand.

Also, the facet Feelings appeared to discern ALS from FTD (27.46 vs 24.22, p=0.031).

Openness to feelings implies receptivity to one's own feelings and emotions and the evaluation of emotion as an important part of life; high scorers experience deeper and more differentiated emotional states and feel both happiness and unhappiness more keenly than others do. Conversely, low scorers have somewhat muted affects and do not believe that feeling states are of much importance.

| Five domains' facets | **FTD**  **(n 27)** | **ALS**  **(n 13)** | **FTD vs ALS**  **(p)** |
| --- | --- | --- | --- |
| Neuroticism |  |  |  |
| N1: Anxiety | 23.40(4.29) | 26.38(5.78) | 0.07 |
| N2: Angry Hostility | 18.85(5.67) | 19.69(4.51) | 0.64 |
| N3: Depression | 19.55(5.04) | 19.84(4.99) | 0.86 |
| N4: Self-Consciousness | 20.33(5.51) | 18.46(6.33) | 0.34 |
| N5: Impulsiveness | 23.74(4.42) | 24.15(5.71) | 0.80 |
| N6: Vulnerability | 20.96(6.17) | 19(3.10) | 0.28 |
| Extraversion |  |  |  |
| E1: Warmth | 28(5.65) | 31(3.24) | 0.084 |
| E2: Gregariousness | 19.33(5.56) | 21.61(3.42) | 0.18 |
| E3: Assertiveness | 22.07(6.33) | 24.15(3.48) | 0.277 |
| E4: Activity | 22.74(5.82) | 26.23(4.18) | 0.061 |
| E5: Excitement Seeking | 21.11(5.00) | 20.23(6.22) | 0.63 |
| E6: Positive Emotions | 23.29(5.09) | 27.30(3.14) | 0.013* |
| Openness |  |  |  |
| O1: Fantasy | 21.07(5.38) | 24.92(4.42) | 0.031* |
| O2: Aesthetics | 21.22(5.01) | 24.38(5.99) | 0.087 |
| O3: Feelings | 24.22(4.28) | 27.46(4.31) | 0.031* |
| O4: Actions | 23.33(4.85) | 25.15(4.59) | 0.266 |
| O5: Ideas | 20.18(5.51) | 22.38(5.48) | 0.243 |
| O6: Values | 23.85(4.20) | 25.53(3.84) | 0.229 |
| Agreeableness |  |  |  |
| A1: Trust | 25.51(6.17) | 25.92(4.53) | 0.83 |
| A2: Straightforwardness | 28(6.00) | 28.07(3.94) | 0.96 |
| A3: Altruism | 29(5.83) | 31.30(4.92) | 0.22 |
| A4: Compliance | 23.37(4.66) | 20.69(5.31) | 0.11 |
| A5: Modesty | 26.07(4.99) | 27.46(4.73) | 0.40 |
| A6: Tendermindedness | 28.44(5.02) | 29.92(3.40) | 0.34 |
| Conscientiousness |  |  |  |
| C1: Competence | 27.70(5.80) | 29(2.97) | 0.45 |
| C2: Order | 27.44(6.18) | 27.92(6.51) | 0.82 |
| C3: Dutifulness | 31.03(5.00) | 31.92(4.15) | 0.58 |
| C4: Achievement Striving | 26.33(5.47) | 28.23(5.35) | 0.30 |
| C5: Self-Discipline | 29.25(6.34) | 31(3.74) | 0.36 |
| C6: Deliberation | 25.03(5.82) | 26.69(4.15) | 0.36 |

**Supplementary Table 1. Five domains’ facets.**

## Evolution of personality traits over time

To evaluate changes over time between premorbid and current personality in the two diagnostic groups, we performed repeated-measures ANOVAs for each personality domain.

There was a significant effect of time on Neuroticism (p=0.005), since it tended to increase from premorbid to current personality in both groups. However, there was no significant interaction between time and diagnostic group (p=0.59), meaning that FTD and ALS patients behaved similarly over time.


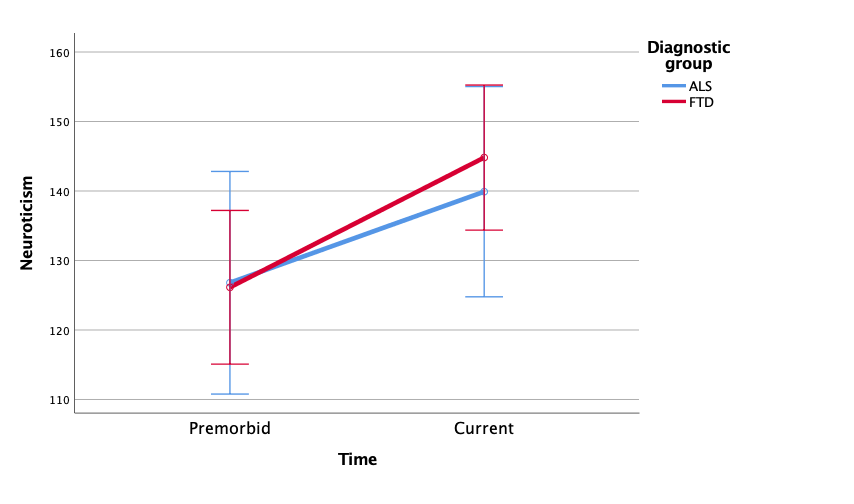


**Supplementary Figure 1.** Evolution of Neuroticism over time between diagnostic groups.

Similarly, in both Extraversion and Openness there was a significant effect of time (p=0.027, p=0.004), in that they tended to decrease in both FTD and ALS patients, but there was no interaction time-by-group (p=0.595, p=0.693). As previously mentioned, there was a significant effect of diagnostic group.

**
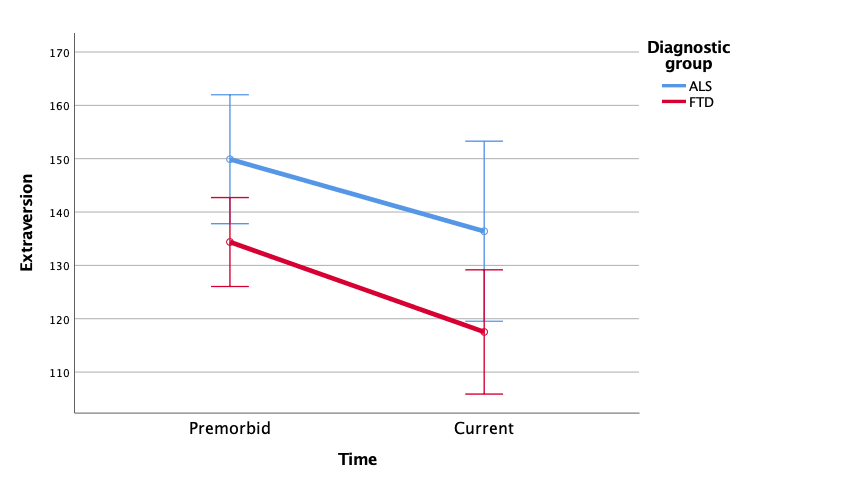
**

**Supplementary Figure 2.** Evolution of Extraversion over time between diagnostic groups.


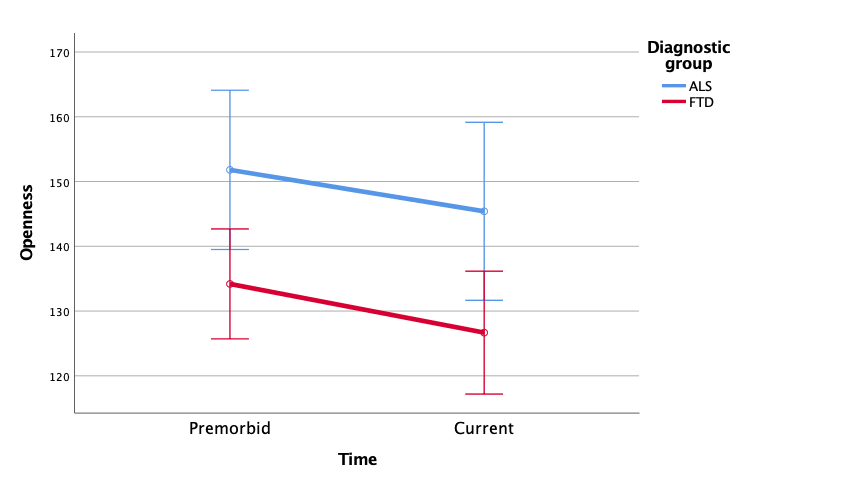


**Supplementary Figure 3** Evolution of Openness over time between diagnostic groups.

Conversely, the two groups behaved differently in the Agreeableness domain, with a significant interaction between time and diagnostic group (p=0.02). In fact, they were similar in premorbid life, but became different in current time, since FTD patients decreased their Agreeableness scores and ALS patients increased them. There was no significant effect of time or diagnosis alone (p=0.305, p=0.2).


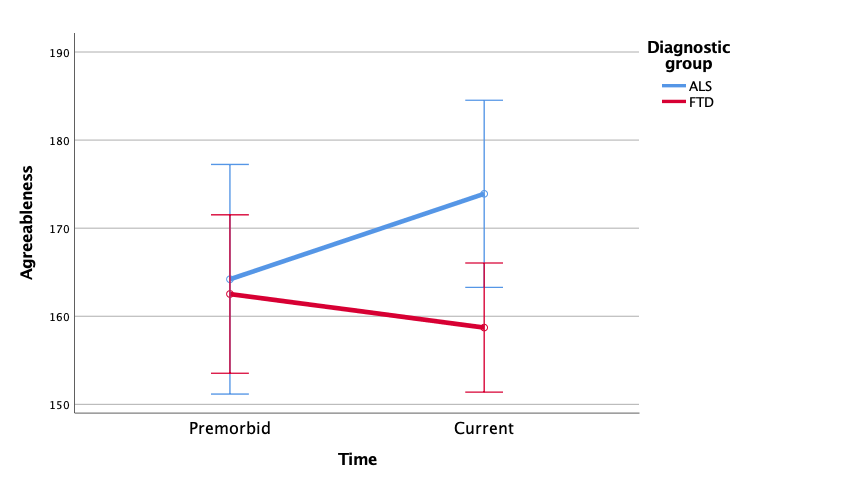


**Supplementary Figure 4.** Evolution of Agreeableness over time between diagnostic groups.

Finally, there was a significant effect of time (p=0.004) and an almost significant interaction time-by-group (p=0.06) in the Conscientiousness domain. In fact, both groups decreased their score during time, but while the reduction was mild for ALS patients, it appeared extremely marked for FTD patients. The effect of diagnostic group also resulted significant (p=0.04).


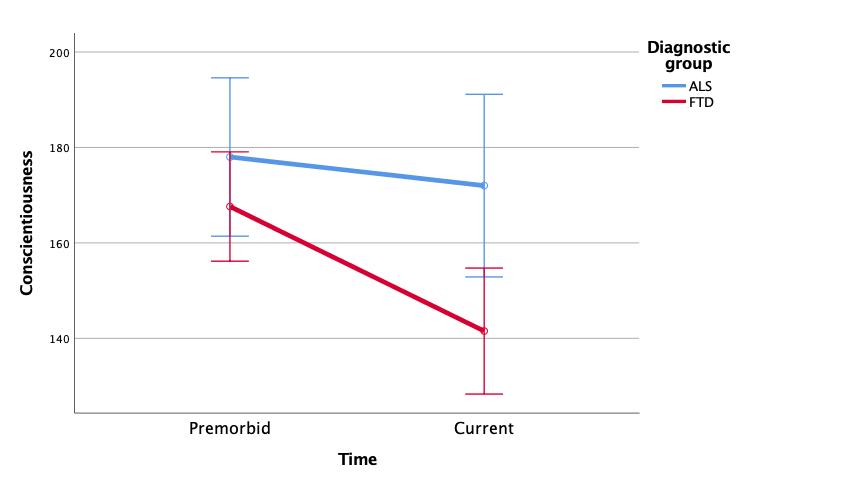


**Supplementary Figure 5.** Evolution of Conscientiousness over time between diagnostic groups.


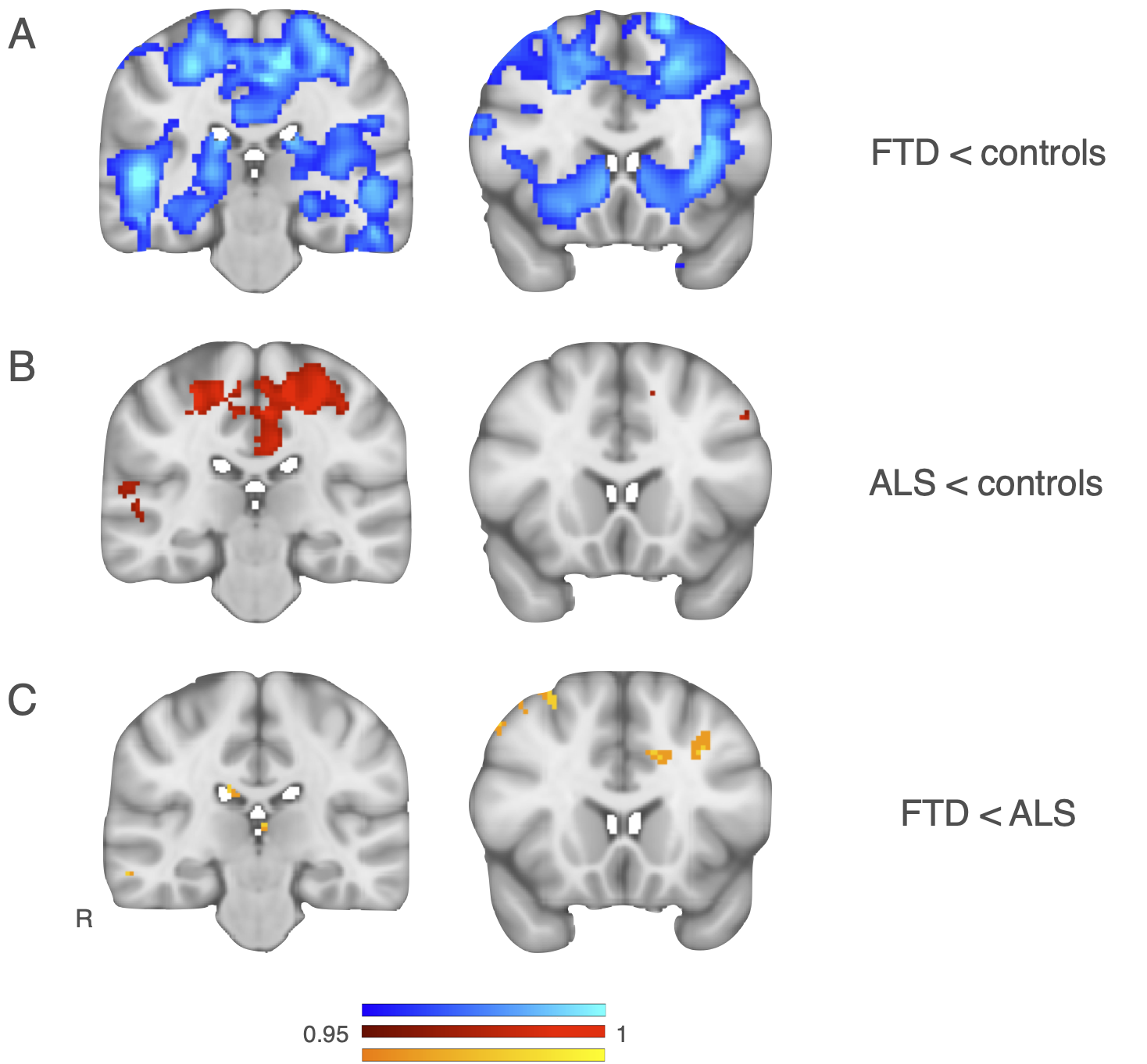


**Supplementary Figure 6.** **Results of VBM analysis, comparison between groups.** A) In blue-light blue, areas of decrease gray matter volume in FTD patients relative to controls. B) In red, areas of decrease gray matter volume in ALS patients relative to controls. C) In yellow-orange, areas of decrease gray matter volume in FTD relative to ALS patients. Results are shown at p _uncorr_ < 0.005 for visualization purposes.

#### Correlation between GM volume and personality traits across all subjects

The VBM correlational analysis with premorbid NEO-PI-3 scores (which also included age, MMSE and current NEO-PI-3 scores as covariates of no interest) showed a significant positive correlation between GM volume and Neuroticism in bilateral hippocampus for left more than right (TFCE corrected p < 0.05) (Fig.7). In detail, the lowest the premorbid score on Neuroticism the greater the atrophy in both hippocampi, left parahippocampal gyrus, and right and left temporal fusiform gyri. A significant positive correlation (TFCE corrected p < 0.05) was also found between GM volumes and Conscientiousness in the right angular gyrus. In the same region, also Extraversion and Openness were found to be positively correlated, but at p<0.005 uncorrected.


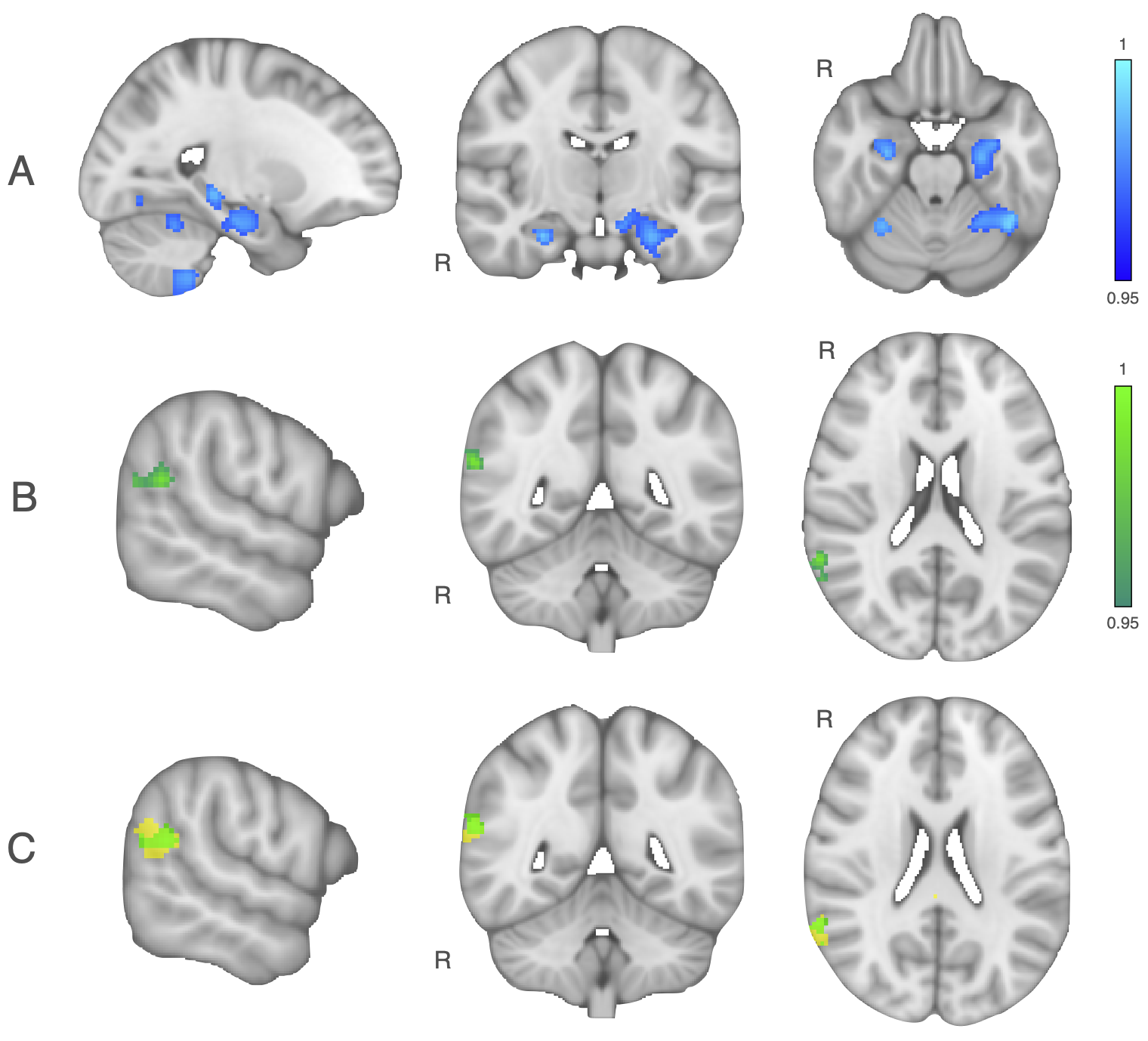


**Supplementary Figure 7.** **Results of the VBM correlational analysis between GM and the five NEO-PI-3 domains across all patients.** A) In blue-light blue, areas of significant correlation between GM and Neuroticism (TFCE corrected p<0.05). B) In green, areas of significant correlation between GM and Conscientiousness (TFCE corrected p<0.05). C) In light green, overlay of areas of positive correlations between GM and Extraversion, Openness and Conscientiousness at p _uncorr_ < 0.005, superimposed on areas of significant correlation between GM and Conscientiousness shown in B.
